# Sensitivity Analysis of the Catalysis Recombination Mechanism on Nanoscale Silica Surfaces

**DOI:** 10.3390/nano12142370

**Published:** 2022-07-11

**Authors:** Lichao He, Zhiliang Cui, Xiangchun Sun, Jin Zhao, Dongsheng Wen

**Affiliations:** 1 School of Aeronautical Science and Engineering, Beihang University, Beijing 100191, China; zanjiachaoshu@buaa.edu.cn (L.H.); by1805107@buaa.edu.cn (Z.C.); sunxch1979@buaa.edu.cn (X.S.); 2 Ningbo Institute of Technology, Beihang University, Ningbo 315100, China; 3 Hangzhou Innovation Institute (Yuhang), Beihang University, Hangzhou 310052, China; 4 Lehrstuhl für Thermodynamik, Technical University of Munich, 80333 Munich, Germany

**Keywords:** surface catalysis recombination, silica surface, reactive molecular dynamics, sensitivity analysis, thermal protection material

## Abstract

A deep understanding of surface catalysis recombination characteristics is significant for accurately predicting the aeroheating between hypersonic non-equilibrium flow and thermal protection materials, while a de-coupling sensitivity analysis of various influential factors is still lacking. A gas–solid interface (GSI) model with a hyperthermal flux boundary was established to investigate the surface catalysis recombination mechanisms on nanoscale silica surfaces. Using the reactive molecular dynamics (RMD) simulation method, the effects of solid surface temperature, gas incident angle, and translational energy on the silica surface catalysis recombination were qualified under hyperthermal atomic oxygen (AO), atomic nitrogen (AN), and various AN/AO gas mixtures’ influence. It can be found that, though the Eley–Rideal (E–R) recombination mechanism plays a dominant role over the Langmuir–Hinshelwood (L–H) mechanism for all the sensitivity analyses, a non-linear increasing pattern of AO recombination coefficient *γ*_O2_ with the increase in incident angle *θ*_in_ and translational energy *E*_k_ is observed. Compared with the surface catalysis under hyperthermal AO impact, the AN surface adsorption fraction shows an inverse trend with the increase in surface temperature, which suggests the potential inadequacy of the traditional proportional relationship assumptions between the surface adsorption concentration and the surface catalysis recombination coefficient for other species’ impact instead of AOs. For the incoming bi-component AO/AN gas mixtures, the corresponding surface catalysis coefficient is not the simple superposition of the effects of individual gases but is affected by both the intramolecular bond energies (e.g., O_2_, N_2_) and intermolecular energies (e.g., Si/N, Si/O).

## 1. Introduction

As the flying speed increases, the extreme aeroheating brings the problem of the “heat barrier”, which is the major barrier for the next generations of high-speed aircraft [1,2]. An in-depth understanding of how aerothermal heat is generated and how to accurately predict the amount of heat produced is of paramount importance for designing reliable thermal protection systems (TPS) [3,4,5].

The high-temperature thermo-chemical non-equilibrium gas flows due to the high speed, i.e., Mach number > 5, cause extremely complicated heterogeneous interactions, such as surface catalysis, oxidation, and ablation, between the gas and TPS materials [6,7,8]. Silica is an important material that has been widely used in non-ablative TPS [9,10], which has the characteristics of thermostability, anti-ablative, and shock resistance [11]. Due to the high-temperature gas effect, the surrounding air may become dissociated into atomic form, i.e., atomic oxygen and nitrogen, whose interactions with the silica surface display remarkable differences from that of molecular impact [12]. For air at 1 atm pressure, oxygen dissociation begins at about 2000 K and is completed at about 4000 K. At that temperature, nitrogen dissociation begins, and N_2_ is essentially totally dissociated at about 9000 K. The air, originally considered to be composed of 79% N_2_ and 21% O_2_, can therefore be assumed as a five-species model (N_2_, O_2_, NO, N and O). The recombination of dissociated atomic oxygen (AO) or atomic nitrogen (AN) due to the high temperature’s effect on their gaseous compounds on the silica surface is a typical surface catalytic exothermic reaction, which significantly affects the prediction of aeroheating [6,13]. For instance, a weak catalytic surface could cause a large decrement in surface heat flux as it prevents the recombination of the dissociated atoms on the surface, hence reducing exothermic heat releases. The determination of the catalytic effect, termed as “recombination coefficient”, becomes crucial [14,15,16].

**Figure 1:**
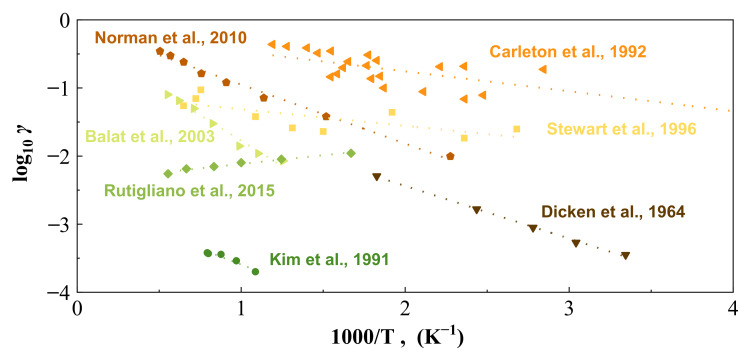
Typical silica surface catalysis recombination coefficient measurement values [23,24,25,26,27,28,55].

Traditional numerical strategies such as Computational Fluid Dynamics (CFD) modeling always employ a constant catalytic coefficient assumption to predict the aerodynamic heating, termed non-catalytic (i.e., *γ* = 0), fully-catalytic (i.e., *γ* = 1), or a finite catalytic assumption [17], which results in a large difference in the estimation of heat flux. It has been shown that the predicted aerodynamic heat could differ 3–4 times [17,18] in the stagnation point under hypersonic reentry flow conditions by using either a non-catalytic or fully-catalytic assumption. Aiming to accurately predict aerodynamic heating, scholars have developed the finite catalytic model [18,19,20]. However, the catalytic recombination coefficient in the finite catalytic model comes from the fitting of the experimental results; the results of different experiments are significantly varied [21,22,23], leading to different simulation results. For instance, the oxygen prepared by Dicken et al. [24] contained a small amount of water molecules, and the –OH ions produced by the ionization of water molecules bonded to the surface sites of the test piece, resulting in the reduction in the surface catalytic recombination coefficient. Stewart et al. [25,26] used emission spectrum atomic diagnosis to obtain atomic concentration, which was based on the assumption that all tracer atoms had made a transition, hence a large discrepancy in the measurement of atomic concentration. Carleton et al. [27] obtained the catalytic recombination coefficient by measuring the atomic O loss, rather than the reaction products. Consequently, other products such as O_3_ may interfere with the accuracy of the catalytic recombination coefficient, leading to a much higher measured catalytic recombination coefficient. Balat et al. [23] conducted the experiments at 200 Pa, but the experimental method was difficult to eliminate the error caused by the O_2_ molecules formed in the gas phase. Considering the large uncertainties among different techniques, it becomes extremely difficult to unify a reliable recombination coefficient from different experimental results, as shown in Figure 1. From a microscopic simulation front, Rutigliano et al. [28] only calculated the E–R reaction mechanism via DFT investigation, so the data they obtained are lower and the trend is different from other conclusions. Consequently, the thermal protection system (TPS) needs to use high design margins to protect the inner structure due to the uncertainty of the recombination coefficient and the lack of mechanistic understanding [17], especially the microscopic phenomena at the gas–solid interface [29]. In this regard, molecular dynamics simulation can provide a promising solution to advance the catalytic reaction mechanism at the microscale [30,31,32].

Recently, an atomistic-scale numerical technique using Reactive Force Field (ReaxFF) potential based on a classical Molecular Dynamics (MD) simulation method, also known as the Reactive Molecular Dynamics (RMD) method, has been proposed to reveal more microscopic features that contribute to the more accurate determination of the catalytic effects [33,34,35]. Quite a few RMD studies have been conducted to investigate the heterogeneous reactions between atomic oxygen impact onto typical TPS materials such as graphene, silica, and different metallic surfaces [36,37,38]. Cui et al. [39,40] studied the effects of the incident angle of AO, energy flux density, the number of graphene layers, and the surface morphology on the ablation, and found that the surface morphology had a significant effect on the ablation rate. Bačová et al. [41] presented an investigation of dynamics relations in thick films of cis-polybutadiene chains placed between rough amorphous silica slabs. The results suggested that the monomeric translational motion parallel to the surface was affected by the presence of the silica slab. Gai et al. [42] established the Pt/O/H reaction force field and derived the theoretical adsorption isotherms of O and H on the Pt slab and nanoparticles by using a GCMC/RMD mixed simulation. Jeon et al. [43] simulated the oxidation process of copper on different surfaces and showed that the initial oxidation was determined by the surface energy via analyzing the growth process of copper oxide and the change of surface morphology. Valentini et al. [44] simulated the adsorption process of O_2_ impacting the Pt slab and observed that the adsorption probability of O_2_ increased with the incident energy in the range of 0.1–0.4 eV of O_2_ incident energy. Chen et al. [45] simulated the early oxidation process of the Si(100) surface and illustrated that oxygen transport was the dominant factor in the initial oxidation process. The formation of the oxide layer would subsequently hinder oxygen transport and prevent further oxidation, and the increase in temperature could promote the migration of oxygen to the deep Si layer. Cao et al. [46] changed the surface dimer density of Si by biaxial tensile strain and revealed that the dimer plays an important role in the surface oxidation reaction by modifying the adsorption amount and penetration depth of O. Newsome et al. [47] established a ReaxFF reaction force field of Si–Si, Si–O, and Si–H, and showed that SiC gradually transformed into silicon oxide and formed graphite-like layers. In the presence of excess O_2_, the graphite-like layer was further oxidized to CO and CO_2_. Gamalo et al. [48] simulated the collision of CO on the surface of preoxygenated β-cristobalite(001), including molecular reflection and non-dissociative molecular adsorption. CO_2_ was formed in a small range by the Eley–Rideal reaction. Mao et al. [49] studied the reaction process of CO on the surface of *β*-SiO_2_ by using RMD and showed that the reaction of CO on the surface of β-SiO_2_ was mainly molecular reflection and non-dissociation adsorption. With the increase in incident energy, the molecular reflection ratio increased, which verified the low reactivity of CO on the surface of β-SiO_2_. Khalilov et al. [50,51,52] studied the growth mechanism of SiO_2_ via atomic and molecular oxygen impacting Si and SiO_2_ surfaces. It was found that the thickness of the oxide layer only depended on the incident energy when the temperature was below 700 K, but became reliant on both incident energy and surface temperature at temperatures over 700 K, due to the increased penetration and diffusion rate of the oxidant at higher temperatures. Yang et al. [53] simulated the reaction process of the O atom and *β*-SiO_2_ surface at different temperatures under the thermal equilibrium of wall and gas at 10 atm. It was found that the catalytic recombination coefficient obtained by RMD was in good agreement with the experimental ones at high temperatures, but was higher at low temperatures.

To calculate the recombination coefficient, a gas–solid model is generally established. Previously, Cozmuta et al. [54] established a gas–solid model for the reaction of O_2_ and N_2_ mixed gases on an SiO_2_ surface and revealed that the adsorption of the O atom on the SiO_2_ surface was stronger than that of the AN atom. The gas column model can calculate the catalytic recombination coefficient, which, however, brought some statistical errors to the products due to the difficulty in ruling out the influence of the interactions among atoms/molecules in the gas phase. For instance, the reflected atoms may become recombined into molecules in the gas phase, which rendered some uncertainties in calculating the catalytic effect. Based on the gas column model of Cozmuta, Norman et al. [55,56,57,58] established a flux boundary model to study the catalytic recombination mechanism and site number of O atoms at different temperatures and pressures on the surface of *β*-SiO_2_. Such a flux boundary model eliminated the interference of gas reactions in the gas phase and increased the statistical accuracy catalytic recombination coefficient. This makes it possible to further analyze the influence of environmental factors such as temperature and pressure on the catalytic reaction.

Such a brief review shows that our current understanding of the impact of hyper-enthalpy atoms on TPS materials is still poor. Mostly single atom impact has been investigated under idealistic conditions based on a gas–solid column model. However, in a real flight, the surrounding gas is a mixture of N and O atoms or their molecule forms, and their contents also vary with different attack angles and flight heights due to different viscous heating effects. Many questions, such as the effects of different gas mixtures, the impact angle, and the impacting energies on the catalytic recombination over TPS materials, especially SiO_2_, remain unanswered. We have recently established a gas–solid interface flux (GSI) model in RMD and successfully revealed the effects of solid surface morphology and impacting gas mixture on graphene and SiC surfaces [39,40], as well as the competing effects between surface oxidation and catalysis [59]. To answer the questions raised above, we will use the established flux boundary model to study the impact of different O/N mixtures under various conditions, including the effects of O / N atoms, incident angle, and incident translational energy, on SiO_2_ surfaces, and reveal their influences on the surface catalytic recombination, an area is of importance for the future design of silicon-based TPS materials.

## 2. Materials and Methods

All RMD simulations in the present work were carried out based on an open-source simulation software LAMMPS (Large-scale Atomic/Molecular Massively Parallel Simulator) [60] developed by Sandia National Laboratories. The ${\mathrm{ReaxFF}}_{\mathrm{SiO}}^{\mathrm{GSI}}$ force field, developed by Kulkarni et al. [61] was applied in the present work and had also been successfully employed to describe the chemistry and dynamics in oxygen–silica gas-surface interactions (GSI) [53,54,56].

**Figure 2:**
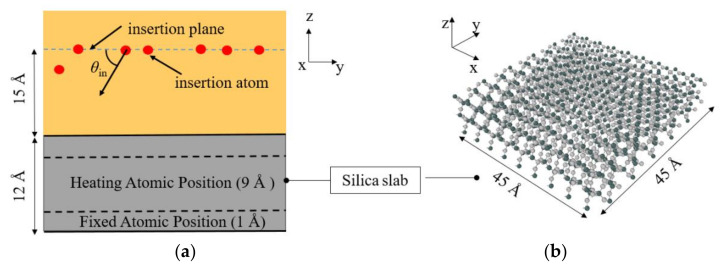
Schematic illustration of gas-solid interaction (GSI) model with flux boundary. (**a**) the RMD simulation configuration for the gas-solid interface (**b**) the silica surface configuration.

A novel modified GSI model was established and extended based on the previous RMD research [54,55] with a modified flux boundary, as shown in Figure 2. Both gas and solid phases were modeled: (a) For the silica substrate (a typical non-ablative TPS material) as the solid phase, an *α*-SiO_2_ plate cleavage along the (001) surface with a thickness of 12 Å was prepared with a planar dimension of 45 × 45 Å^2^ in the *x*- and *y*-axis directions, respectively. To prevent the surface from moving downwards during the continuous gas collisions, the last layer of all the atoms for the silica slab was fixed. Moreover, apart from the atoms in the surface top-two atom layers, all the remaining atoms for the silica slab were heated and equilibrated with the Langevin thermostat [62], ensuring the temperature stability of the silica surface temperature during the bombardment of the impinging gas. (b) For the gas phase in this modified GSI model, a single oxygen or nitrogen atom was generated every 1.0 ps from a random horizontal position at a height of 15 Å above the surface with a prescribed translational energy *E*_tr_ and incident angle *θ*_in_. The pressure at the micro/nano-scale gas–solid interface inside the boundary layer is very low during the hypersonic flow conditions, which can be assumed as a one-by-one atom bombardment process for simulations to decouple the complicated thermal/mechanical/chemical interactions. It should be noticed that for the traditional GSI model, the *z* dimension of the simulation box was designed as long as 240 Å to prevent further recombination in the gas phase after collisions at the gas–solid interface [39]. However, the possible interactions and recombination as gas–gas reactions within this long gas column may lead to potential uncertainties or errors in properly identifying and evaluating the gas–surface interactions. To tackle this issue, a novel GSI model was modified with a flux boundary for the impinging gas, where all the species located 15 Å above the silica surface were analyzed every 1000 fs and then deleted to avoid further gas–gas interactions within the long gas column.

The silica surfaces for all the simulations were relaxed for 50 ps using the Nose/Hoover thermostat to achieve thermal equilibrium towards the target temperature with a time step of 0.25 fs before inserting any gaseous species onto the solid slab, then followed by simulation with a period of 1000 ps (To run a 1000-ps simulation took about 60 h with 16 processors). Reflective and periodical boundary conditions were employed in the directions parallel and perpendicular to the surface, respectively. To identify the surface temperature *T*_s_, gas incident angle *θ*_in_, translational energy *E*_k_, and mixture fraction effects on the silica surface catalysis recombination performance under both hyperthermal AO and AN flux bombardment, 38 models were analyzed in this work, whose detail parameters are shown in Table 1.

Since the material surface catalysis recombination coefficient is considered one of the most significant parameters, the evaluation results of the present work are calculated using the following formula:

(1) $$\gamma =\frac{\mathrm{Flux}\;\mathrm{of}\;\mathrm{atoms}\;\mathrm{recombining}\;\mathrm{at}\;\mathrm{the}\;\mathrm{surface}}{\mathrm{Flux}\;\mathrm{of}\;\mathrm{atoms}\;\mathrm{impinging}\;\mathrm{on}\;\mathrm{the}\;\mathrm{surface}}$$

which is defined as the ratio of the impinging atoms that recombine at the surface over the total impinging atoms on the surface. It is notable that the Eley–Rideal (E–R) and Langmuir–Hinshelwood (L–H) recombination mechanisms are the two dominant mechanisms in the surface catalysis recombination process [63]. The E–R recombination mechanism refers to the recombination process in which the incoming oxygen atom, when hitting an adsorbed atom onto the surface, recombines into a molecule and then desorbs from the surface. In the L–H recombination mechanism, the molecule formation is caused by surface diffusion, enabling two atoms adsorbed on the surface to encounter each other for recombination. In order to better describe the surface catalytic effect, according to Deutschman’s research, the main surface reactions of AN/AO mixture gas are listed in Table 2. The symbol “(s)” denotes a free surface site and species with a label “(s)” are adsorbed at the surface. An analysis code was further developed in this work to provide a detailed E–R and L–H recombination mechanism distinction through tracking the molecular oxygen generation pathways.

## 3. Results and Discussion

### 3.1. Validation and Temperature-Dependent Silica Surface Catalysis Characteristics

To demonstrate the validity of the applied ${\mathrm{ReaxFF}}_{\mathrm{SiO}}^{\mathrm{GSI}}$ force field and calculation setup for the RMD simulations, the benchmark cases of the silica surface catalysis characteristics with variations of surface temperature are established and compared with both the reported experimental and simulation results [23,24,26,55] in this section. Under the same AO gaseous phase incident conditions with a translational energy of 0.05 eV normal to the silica slab, four GSI models with flux boundaries are built up under four silica surface temperatures *T*_s_, i.e., 500 K, 1000 K, 1500 K, and 2000 K, respectively.

**Figure 3:**
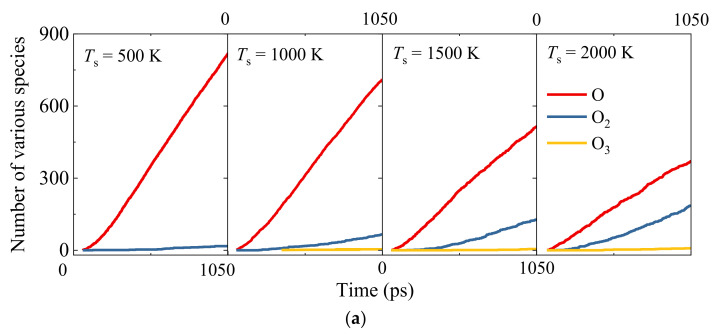

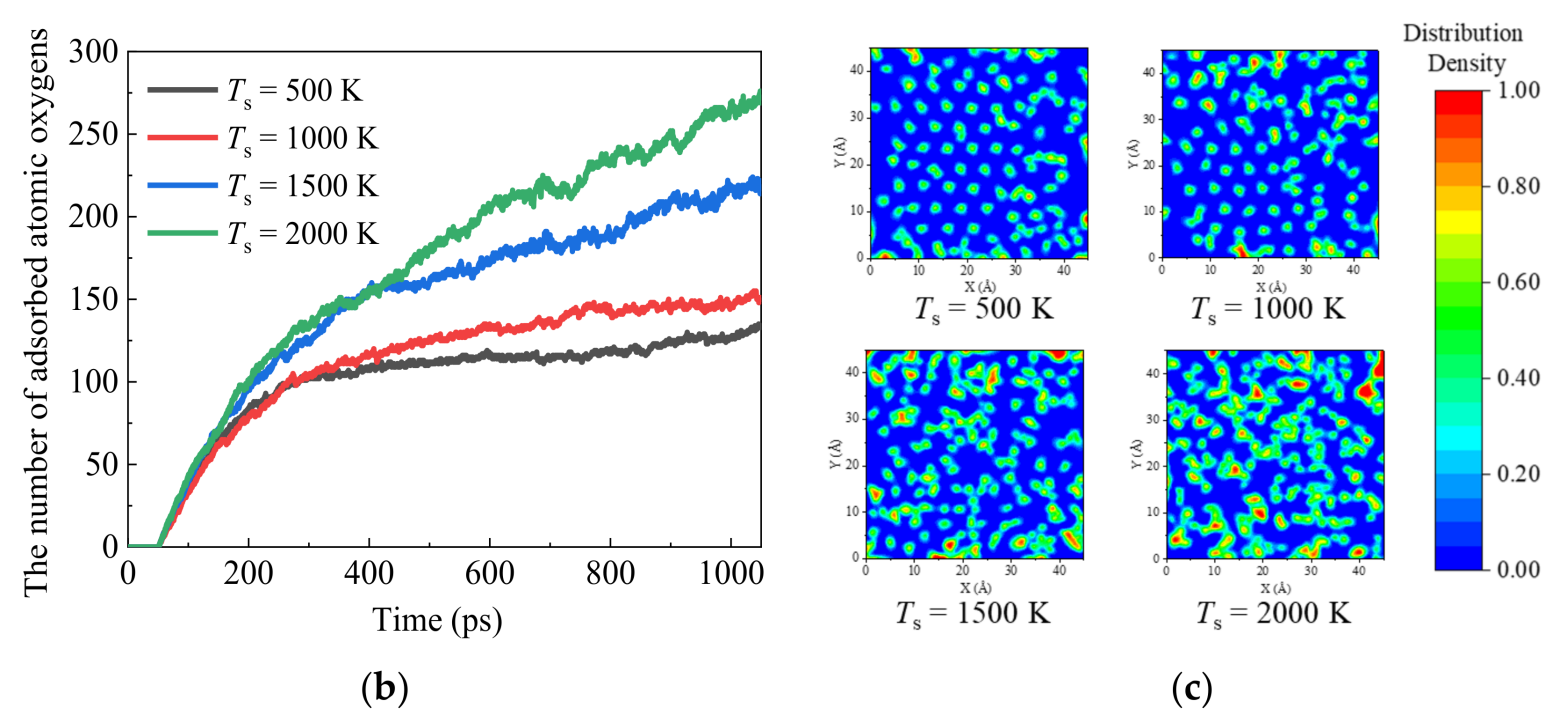
Surface adsorption of oxygen atoms for Models 1–4. (**a**) The number of various species in the gaseous phase, (**b**) the number of oxygen atoms adsorbed with variations of time, and (**c**) the effects of silica surface temperature on the saturated oxygen-atom density distribution.

Both the transient flow-field analysis during the AO continuous bombardment and material response analysis are identified as shown in Figure 3. It can be observed that except for the slight amount of O_3_ molecules, the molecular oxygen O_2_ dominates the compositions of all the gaseous products. It also demonstrates that the heterogeneous surface catalysis recombination reaction is playing the leading role during the gas–surface interactions [O + O → O_2_]. Moreover, with the increase in silica surface temperature *T*_s_, the molecular oxygen O_2_ generation rate becomes larger. This can be explained by a detailed material response analysis for the silica slabs with an insight into the AO surface adsorption conditions, as shown in Figure 3b,c. There is no big change in the amount of O_3_ molecule generation due to the fact that the O_3_ molecule has longer and weaker bonds than the O_2_ molecule. Therefore, it only appears as the intermediate product, which has high energy barriers as an unstable product in a negligible amount. Figure 3b shows that for all the simulation cases, the adsorption rate of monatomic oxygen atoms on the surface of the SiO_2_ slab is quite high at the early stage to around 300 ps, followed by smoothing out the distinguishing spike due to the saturation of the active sites for the silica surface. Moreover, a larger surface coverage fraction of oxygen adsorption is found for the silica surface model with higher equilibrium temperature, which can also be visualized as presented in Figure 3c, implies the higher possibilities of surface catalysis recombination reactions with both L–H and E–R recombination mechanism pathways.

**Figure 4:**
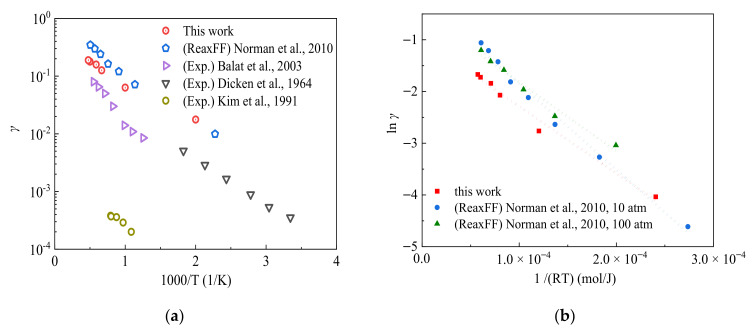
Comparisons of calculated surface catalytic recombination coefficient and the activation energy by Models 1–4. (**a**) The calculated surface catalysis recombination coefficient [23,24,26,55], and (**b**) the calculated activation energy [55].

As shown in Figure 4a, the silica surface temperature-dependent recombination coefficients obtained by RMD simulation results show an exponential trend, which is comparable to the RMD results obtained by Norman et al. [55]. The activation energy of the surface catalysis reactions is further obtained by the linear fitting of the logarithm of the catalytic recombination coefficient with variations of the reverse of surface temperature, as shown in Figure 4b. The quantified activation energy from RMD simulations is compared with both reported experimental and simulation values, as presented in Table 3. It can be found that the activation energy value for surface catalysis reactions obtained by RMD simulation methods is slightly lower than those from experimental measurements. This can be explained by the fact that for RMD simulations, the silica surface is cleaved along the (001) plane, leaving a large number of high-energy suspended Si atoms on the skin layer. Meanwhile, under the experimental conditions, the Si atoms can be oxidized rapidly or form a Si-OH bond with water as it is exposed to air, leading to a reduction in the surface catalytic recombination coefficient value of the SiO_2_ surface.

**Figure 5:**
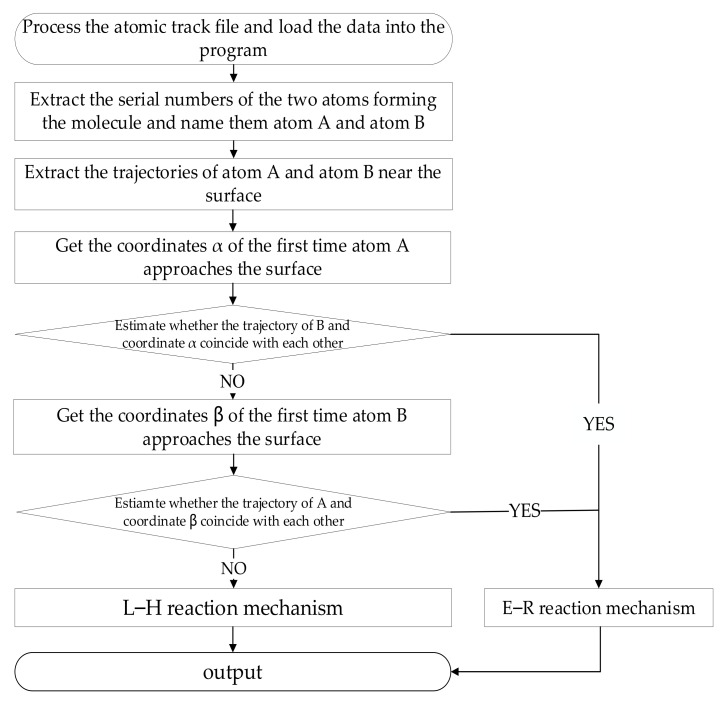
Statistical flow chart of surface catalysis recombination mechanism analysis.

To identify the molecular oxygen generation pathways due to the surface catalysis characteristics, further efforts are made to provide a detailed E–R and L–H recombination mechanism distinction using an own-developed code with its working flow chart, as shown in Figure 5. The transient trajectories of all atomic oxygens recombined with the molecular oxygens are captured at the gas–solid heterogeneous interface (within a height of 1.5 Å from the silica surface [54]). The E–R surface catalysis recombination mechanism is classified if monatomic oxygen collides with another oxygen atom adsorbed onto the silica surface by tracing oxygen history trajectories, otherwise, the L–H mechanism is considered.

**Figure 6:**
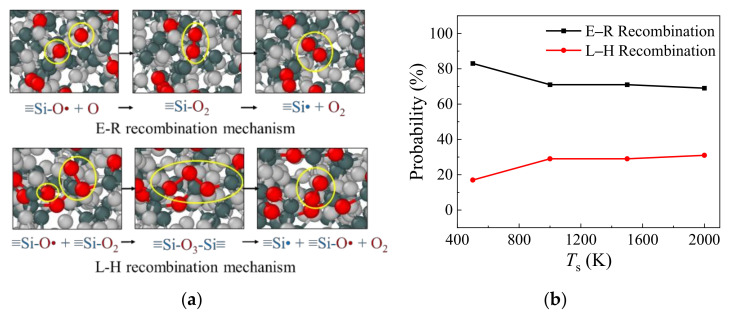
Surface catalysis recombination mechanism analysis by Models 1–4. (**a**) Illustration snapshots for the E–R and L–H recombination mechanisms, and (**b**) the effect of surface temperature on the surface catalysis recombination mechanisms.

The chemical reaction pathways of the surface catalysis phenomenon can be clearly visualized, as shown in Figure 6a, with an easy identification of the classic E–R and L–H recombination mechanisms. (a) The adsorption process: the adsorption behavior of the impinging oxygen atoms happens at the early stage, forming ≡Si–O• sites onto the silica surfaces. (b) The recombination process: colliding with another incoming high-enthalpy oxygen atom, an ≡Si–O_2_ group is formed from the original ≡Si–O• site which is known as a key feature in the E–R recombination mechanism. In addition, the recombination due to the diffusion between two adjacent ≡Si–O• sites may also lead to the reformation of the ≡Si–O_2_ group, which is known as the L–H recombination mechanism. The similar diffusion L–H recombination behavior is also visualized between ≡Si–O• and ≡Si–O_2_ groups, reforming an ≡Si-O_3_-Si≡ group as an intermediate. (c) The desorption process: the chemical bonds in the functional groups between the Si and O atoms break and the O_2_ molecule is generated and released from the silica surface.

Through identifying the molecular oxygen gas generation pathways, the E–R surface catalysis recombination mechanism is found to account for the dominant proportions during the continuous bombardment of monatomic oxygens under various temperature conditions, as shown in Figure 6b. The O_2_ molecule formation originated from the E–R recombination mechanism is measured as high as 83% for the surface temperature *T*_s_ equilibrated at 500 K. With the increase in the silica surface temperature *T*_s_ to 2000 K, the surface coverage fraction of AO becomes greater due to the larger number of active sites on the silica surface at elevated temperature, enhancing the percentages of L–H recombination reactions with diffusion recombination mechanisms for molecular oxygen formations.

### 3.2. Effects of AO Incident Angle and Translational Energy

Considering that the incident AO angle is not always perpendicular to the surface and the incident energy also varies with the speed, six sets of RMD calculations are carried out to investigate the AO incident angle effect on the silica surface catalysis characteristic in a range of angles of attack from 15° to 90° under different translational energy impact.

#### 3.2.1. The Effect of AO Incident Angle

**Figure 7:**
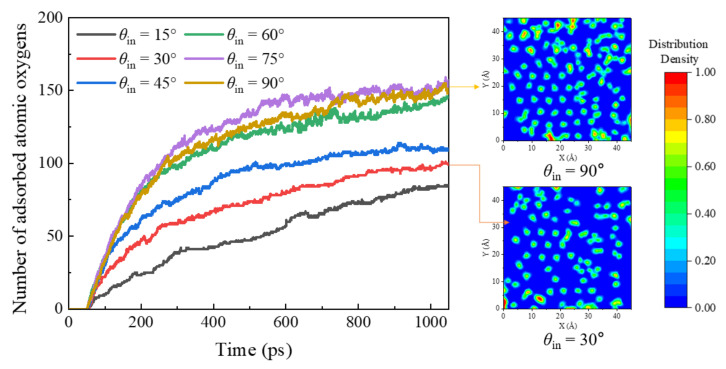
The effect of AO incident angle on the surface adsorption situation with snapshot illustrations of the saturated silicon dioxide surface by Models 1 and II-5–9.

Figure 7 demonstrates the effect of monatomic AO incident angle on the surface adsorption situation with snapshot illustrations of the saturated silicon-dioxide surface. It indicates that the first step of heterogeneous recombination involves the probability of AO adsorption during a gas-phase collision at a clean surface site, where AO acts as the oxidizer. Transient surface adsorption profiles with various AO incident angles imply that the AO adsorption rate is quite large at the early stage, followed by a flattening out due to the saturation of adsorption on active sites of the silica surface. Moreover, this adsorption process is found to be not only temperature-dependent but also incident-angle-dependent, with a comparative observation that with a high angle of incidence, i.e., *θ*_in_ = 90°, the surface coverage fraction of AOs is significantly larger than that with a small angle of incidence, i.e., *θ*_in_ = 30°.

**Figure 8:**
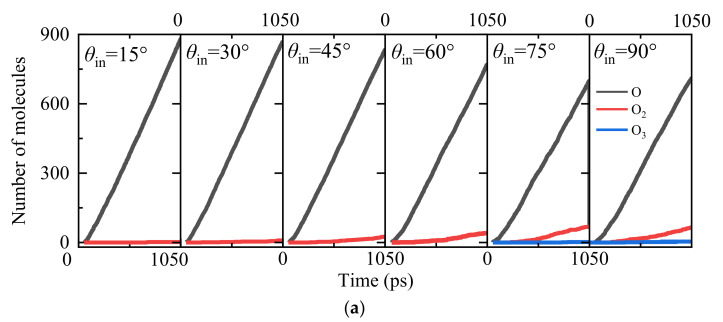

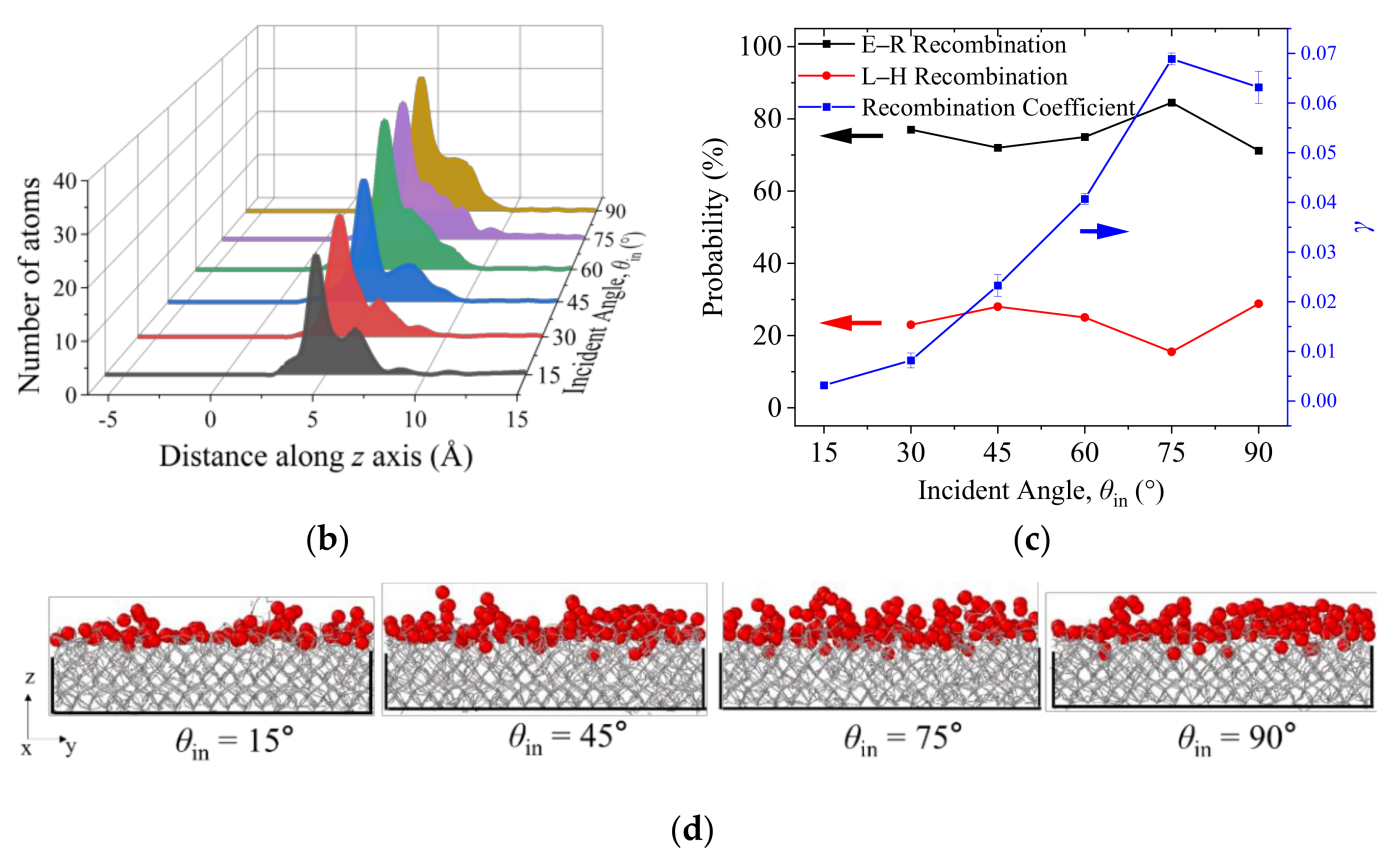
The effect of oxygen incident angle on the flow field, material response, and the silica surface catalysis characteristics by Models 1 and II-5–9. (**a**) The statistics of various species in the gaseous phase during the collisions, (**b**) the incident angle effect on the saturated z-density profiles of oxygen atoms on the silica surface, (**c**) the calculation of surface catalysis recombination coefficient and the detailed recombination mechanism analysis, and (**d**) snapshots for the final AO surface adsorption status.

Figure 8 further quantifies the AO incident angle effect on the gas flow field, silica material response, and the surface catalysis recombination characteristics, and three observations are found. (I) Generally speaking, with the increase in incident angle *θ*_in_, the generated O_2_ molecules increase due to the surface catalysis effect, while at the same time, more AO atoms in the flow field are consumed, so the number of AOs is reduced, as shown in Figure 8a. Taking the incident angle *θ*_in_ of 15° as an illustration, due to its small vertical velocity component perpendicular toward the silica surface, both the fraction of collisions that have sufficient energy to react and the fraction of sufficiently energetic collisions that actually react for adsorption, recombination, and desorption are reduced. Therefore, though carrying the same incoming translational energy, it is more difficult for AOs with a smaller incident angle *θ*_in_ to complete the surface catalysis recombination reaction. (II) The surface catalysis recombination coefficient *γ* is sensitive to the incident angle in a non-linear increasing trend with the increase in AO incident angle *θ*_in_, as shown in Figure 8c. (III) A peak value *γ* of 0.068 is observed to occur when the incident angle *θ*_in_ is around 75° (rather than 90°), which is consistent with the lowest final saturation state of z-density profiles of AOs adsorbing at the silica interface, as shown in Figure 8b, where it indicates the highest adsorption rate for AOs occurring at *θ*_in_ ~ 75°. This observation shows a good agreement with the results from Norman et al. [58] and suggests that there is an optimal relationship between the incident angle and translational energy to achieve the highest surface catalytic recombination rate.

#### 3.2.2. The Effect of Incoming AO Translational Energy

To further identify the effect of incoming AO translational energy on the surface catalysis recombination characteristics, eight models of incoming AO bombardment with various AO incidence translational energy *E*_k_, i.e., from 0.01 to 4.00 eV, are established with a constant surface temperature *T*_s_ of 1000 K.

**Figure 9:**
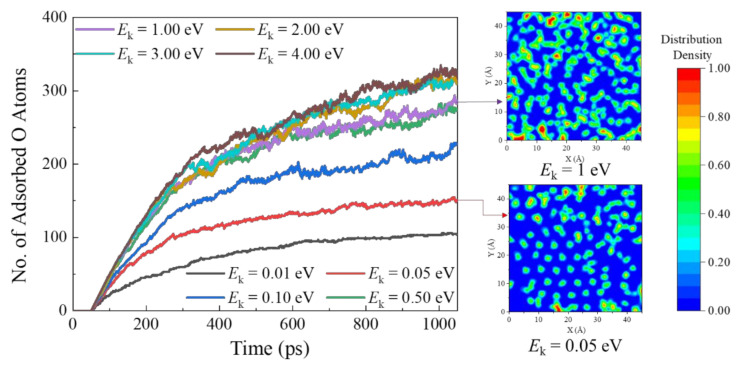
The effect of monatomic oxygen incident translational energy on the surface adsorption situation with snapshot illustrations of the saturated silicon dioxide surface by Models 1 and III-10–16.

The transient surface adsorption profiles for the silica slab under the impact of AOs with various incident translational energy are presented in Figure 9. It can be similarly observed that the AOs initially adhere at the activation sites on the surface, followed by the silica surface adsorption gradually reaching its saturation state. Combining with the final saturated silica surface adsorption contours captured at the gas–solid interface, the AO adsorption saturation state on the silica surface with larger incident translational energy *E*_k_ (e.g., 1.00 eV) is much denser than that with smaller *E*_k_ (e.g., 0.05 eV). This is due to the fact that the increase in AO incident translational energy may provide sufficient energy for the adsorption and oxidation reactions after surface collisions. In addition, it can be found that the surface adsorption saturation state becomes independent of the incident translational energy in the range of 1.00–4.00 eV.

**Figure 10:**
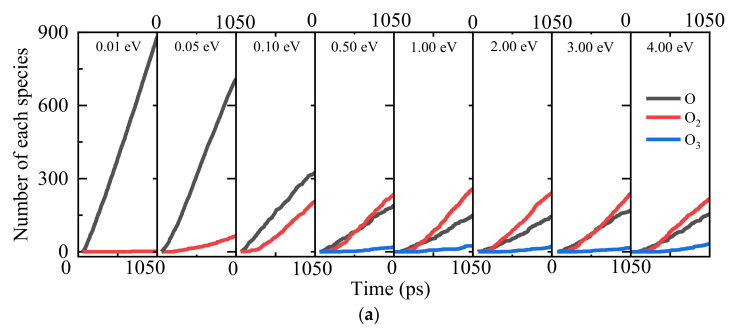

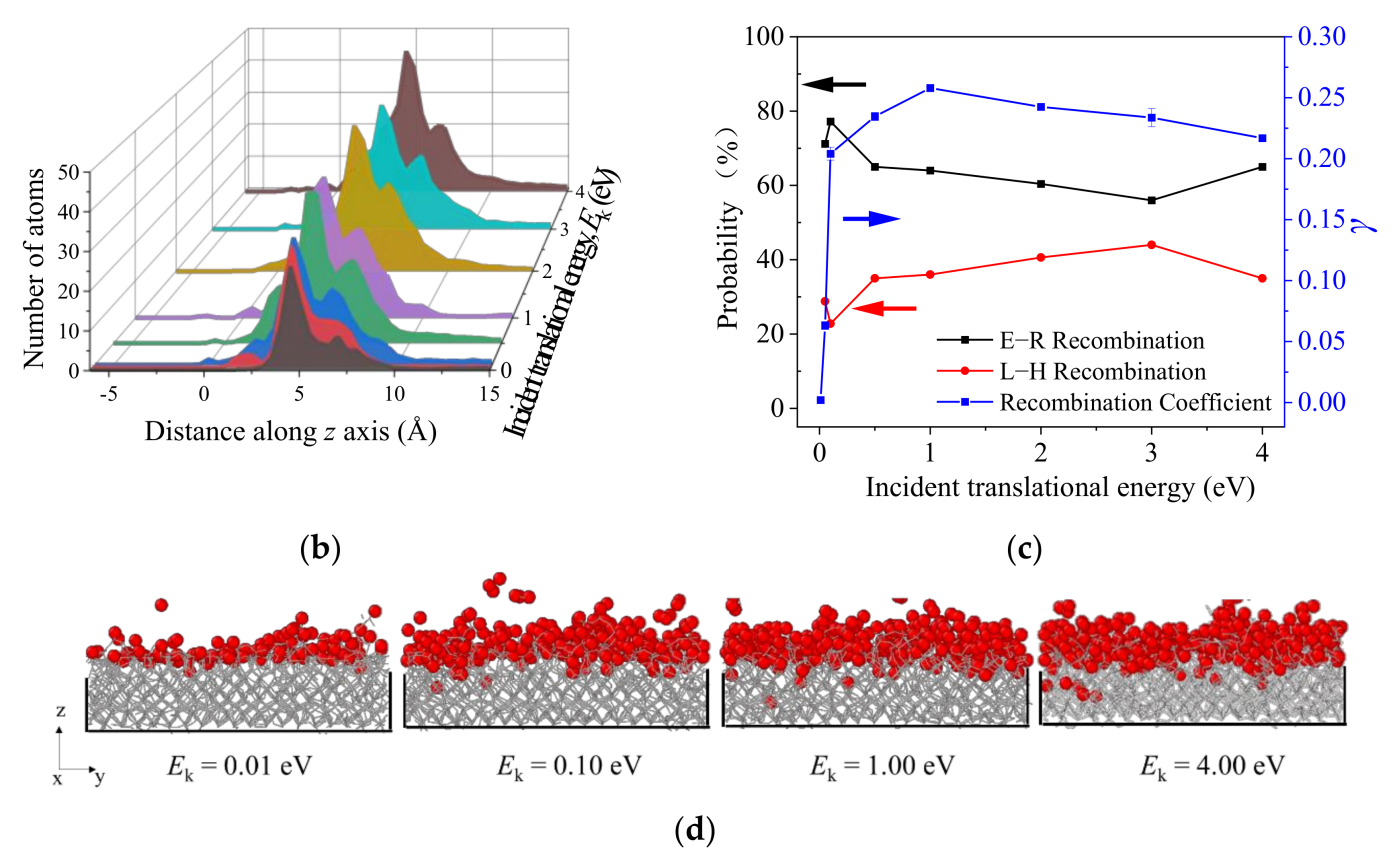
The effect of AO incident translational energy on the flow field, material response, and the silica surface catalysis characteristics by Models 1 and III-10–16. (**a**) The statistics of various species in the gaseous phase during the collisions, (**b**) the incident angle effect on the saturated z-density profiles of oxygen atoms on the silica surface, (**c**) the calculation of surface catalysis recombination coefficient and the detailed recombination mechanism analysis, and (**d**) snapshots for the final AO surface adsorption status.

The effect of AO incident translational energy on the flow field, material response, and the silica surface catalysis characteristics is analyzed in Figure 10. Through tracking the flow field component during the AO continuous impact as presented in Figure 10a, the number of molecular oxygens increases with the increase in AO incident translational energy due to the surface catalysis recombination reactions; correspondingly, the number of AOs in the flow field decreases. It should be noted that when the incident translational energy is greater than 1.00 eV, small quantities of O_3_ molecules are observed. The denser surface adsorption saturation and deeper surface erosion state for the silica slab under the impact of AO carrying larger incident kinetic energy can be visualized in the z-density profiles as shown in Figure 10b. The impact of AOs carrying higher kinetic energy may cause some incident oxygen atoms to break through the silicon–oxygen bonds in the silica surface layer and embedding in-depth into the silicon dioxide plate, eroding the silica and damaging the internal structure of the silica surface, as visualized in Figure 10d.

As a result, the silica surface catalysis recombination coefficient variation as a function of AO incident translational energy is shown in Figure 10c. A continuous growth of *γ* from 0.002 to its peak value of 0.258 is obtained when the AO incident translational energy increases from 0.01 eV to 1.00 eV. A slight decline in *γ* is noticed when *E*_k_ is further increased from 1.00 eV to 4.00 eV, where the same E–R surface catalytic recombination shows as the dominant mechanism during the formation process of O_2_ molecules. This can be explained by two reasons: (1) the incident AOs with high *E*_k_ greater than 1.00 eV tend to embed onto the silica surface due to too-high kinetic energy and directly bounce away from the surface without participating in the recombination reactions. (2) The small amount of unstable reaction-product O_3_ molecules tend to be generated when there are incident AOs carrying kinetic energy greater than 0.50 eV, which leads to the competing effect for free AOs to further form O_2_ molecules, resulting in a slight reduction in the *γ* value with the increase in incidence translational energy greater than 1.00 eV.

### 3.3. The AN Gas Effect on the Silica Surface Catalysis

It has been recognized that under the high-enthalpy (>8.5 MJ/kg) aerodynamic environment, reactions occur in the shock layer and molecular oxygen is almost fully dissociated at the shock. The atomic oxygen is slightly below the equilibrium value because some oxygen is incorporated into NO. The relative amount of N, O, NO, N_2_, and O_2_ at the gas–solid interface, therefore, varies on a case-by-case basis. To separate the behavior of N and O recombination on the silica surface at high temperature, a GSI model with a flux boundary for AN colliding with the SiO_2_ surface is established in this session, and the effects of silica surface temperature, incident angle, and incident translational energy on the surface catalysis recombination phenomenon are investigated in-depth in this session.

**Figure 11:**
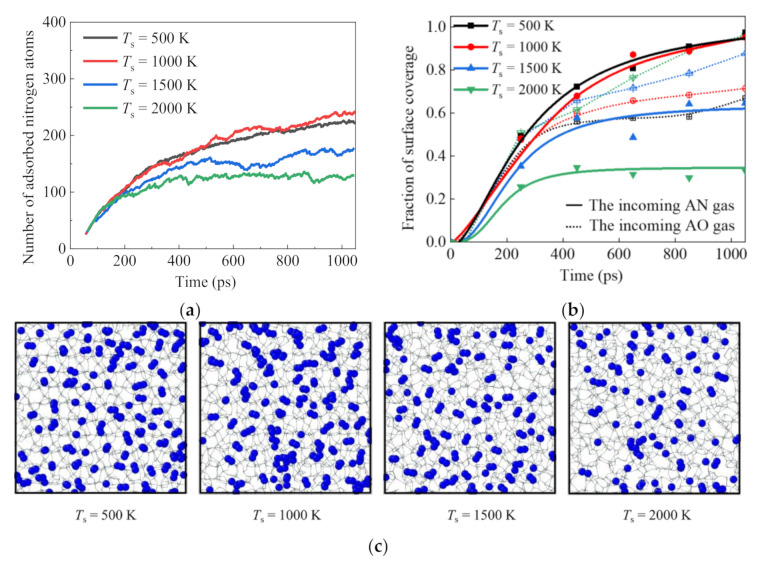
Adsorption of nitrogen atoms on the silica surface with variations in time for the case with the incoming AN gas (Models IV-17–20): (**a**) the effect of surface temperature on the surface adsorption, (**b**) the effect of surface temperature on the surface coverage fraction, and (**c**) snapshots for the final AN surface adsorption status from the view of the x–y plane.

It has been identified that the surface catalysis recombination characteristics are closely dependent on the surface adsorption behaviors during the vertical impact of incoming dissociated gas [55]. Therefore, with an incident angle of 90° and an incident translational energy of 0.05 eV as the benchmark values, the effects of silica surface temperature on the transient surface adsorption status for the AN incidence as the gas phase toward the silica surface are analyzed in Figure 11. Compared with the AO impact, as shown in Figure 3, the AN surface adsorption fraction shows an inverse trend with the increase in surface temperature. The atomistic scale reason implies its close revelations with the bond energy difference among various species, where the intramolecular bond energy for N_2_ gas molecules (~942 kJ/mol) is much higher than the Si–N bond energy (~355 kJ/mol). Therefore, more adsorbed nitrogen atoms would be removed with the increase in the generated N_2_ gas molecules at higher surface temperatures. Conversely, the sparser AN surface adsorption may inversely restrain the surface catalysis recombination property. This extraordinary phenomenon suggests the potential inadequacy of the traditional proportional relationship assumptions [55] between the surface adsorption concentration and the surface catalysis recombination coefficient, as shown in Figure 11a, especially for species other than AO (where the bond energy impact is drowned out for AO incidence cases because the intramolecular bond energy for O_2_ gas molecules (~497 kJ/mol) is very close to the Si-O bond energy (~452 kJ/mol)).

**Figure 12:**
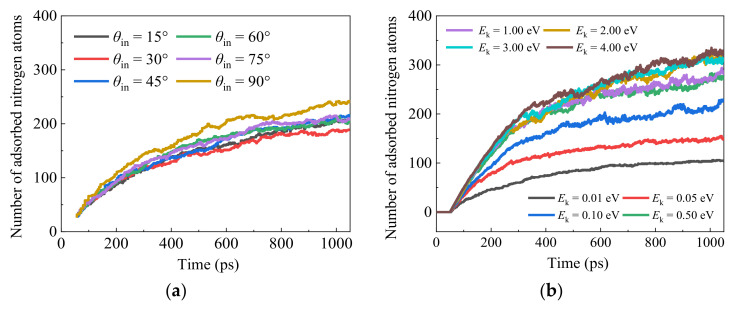
Adsorption of nitrogen atoms on the silica surface with variations in time for the case with the incoming AN gas. (**a**) The effect of incident angle on the surface adsorption by Models IV-21–26, and (**b**) the effect of incident translational energy on the surface by Models IV-27–34.

**Figure 13:**
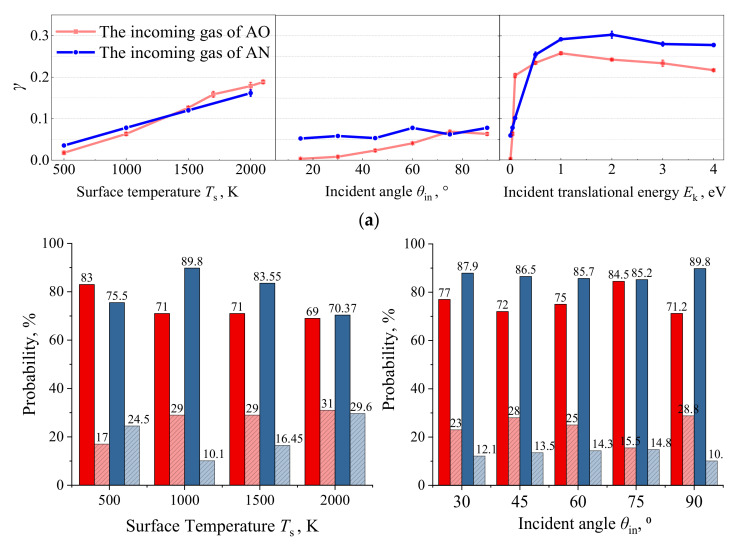

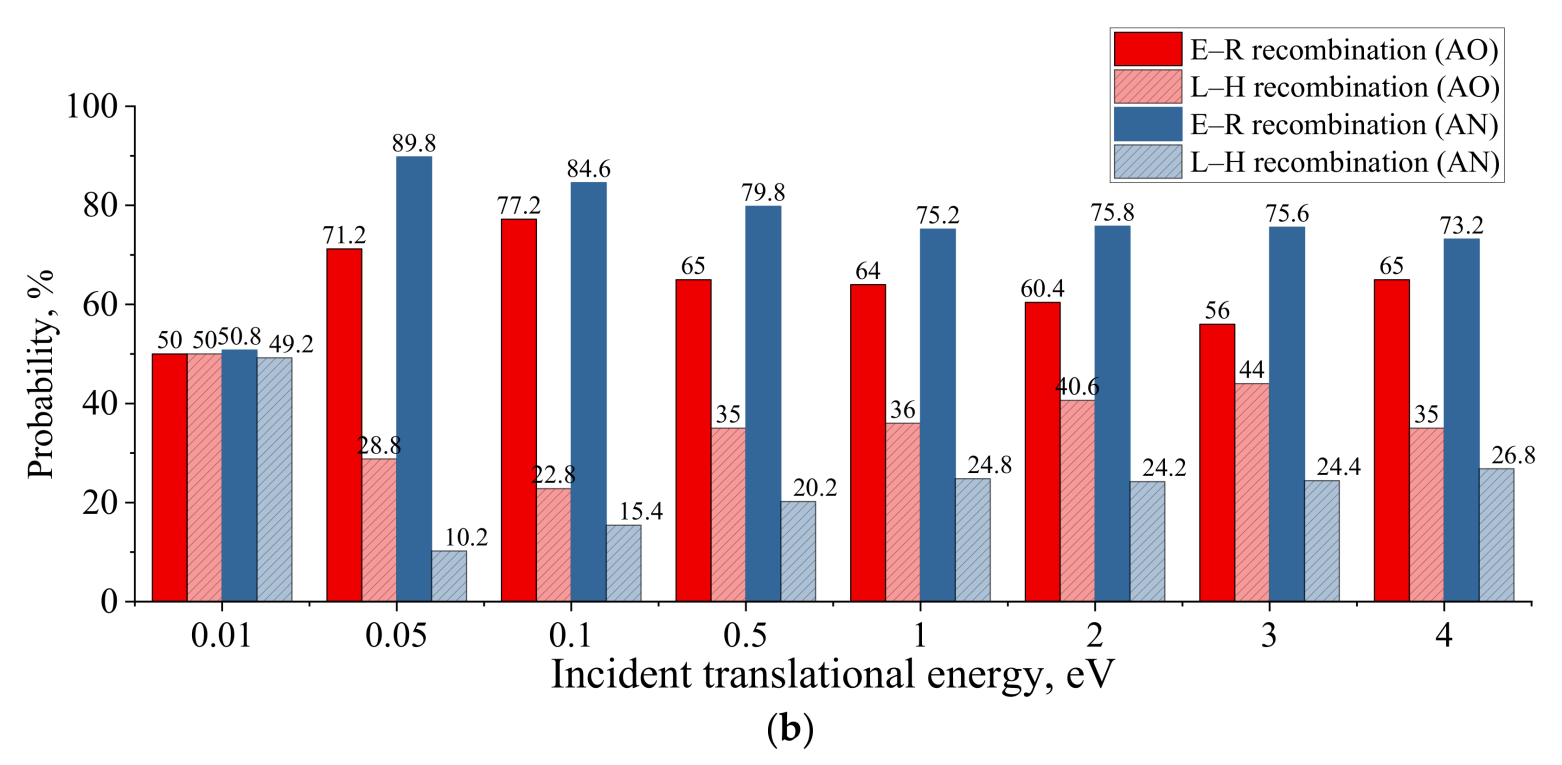
Silicon dioxide surface catalytic recombination coefficient and reaction mechanism analysis for the case with the incoming gas containing full AN (Models IV-17–34). (**a**) Surface catalysis recombination coefficient calculations. (**b**) Recombination mechanism analysis.

In addition, the effects of AN incident angle and incident kinetic energy on the transient surface adsorption situations are analyzed in Figure 12. Combined with the calculated surface catalysis recombination coefficient, as shown in Figure 13a, a similar sensitivity response can be found compared with AO incidence scenarios. (I) A non-linear trend of *γ* is revealed for both incident angle *θ*_in_ and incident kinetic energy *E*_k_ effects. (II) A larger influential magnitude of *E*_k_ is observed for AN surface catalysis performance compared with the *θ*_in_ impact. The corresponding AN recombination mechanism for all the investigated sensitive factors is quantified at the nanoscale and shown in Figure 13b by comparisons with that of AO. A similar E–R recombination type is found to be dominant for AN bombardment toward the silica material surface.

### 3.4. Effects of Bicomponent AO/AN Gas Mixture

To further identify the multicomponent gas effect on the surface catalysis recombination characteristics, a gaseous mixture containing both oxygen and nitrogen atoms is considered as the incidence gas with four various quantitative proportions *f*_O/mixture_ = 75%, 50%, 25%, and 0% (full atomic nitrogen incidence), and compared with the standard benchmarking model that contains 100% AO as the incoming gas.

**Figure 14:**
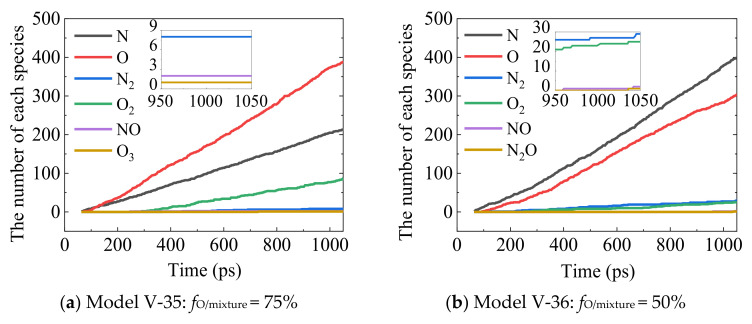

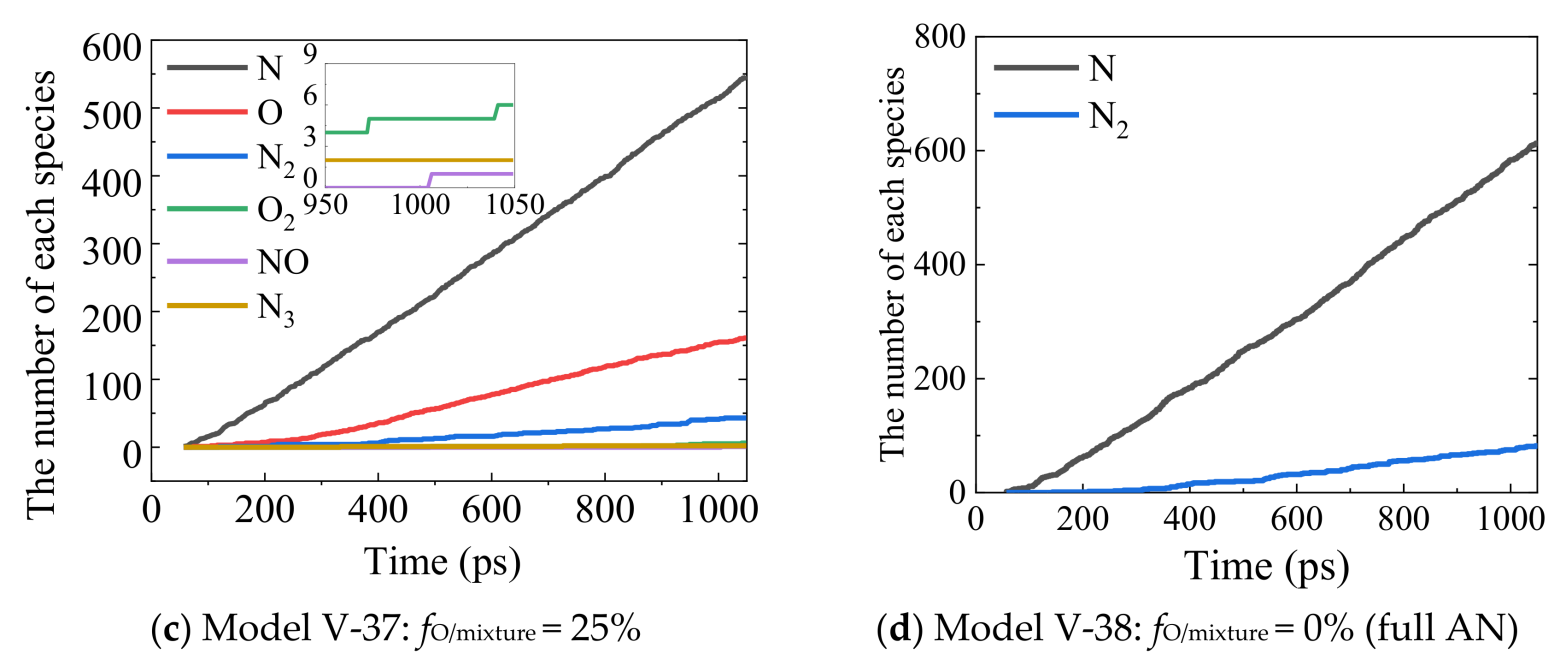
Surface catalytic reaction products. (**a**) Model V-35: *f*_O/mixture_ = 75%, (**b**) Model V-36: *f*_O/mixture_ = 50%, (**c**) Model V-37: *f*_O/mixture_ = 25%, and (**d**) Model V-38: *f*_O/mixture_ = 0% (full nitrogen).

The gaseous generation components during the continuous bombardment at the gas–solid interface for silica surface are analyzed as shown in Figure 14. It can be noticed from the main reaction products for the AN/AO mixture impingement that only a few nitric oxide molecules are found among all the gas-phase products. The gaseous productions for all the models are dominated by the molecular oxygen and nitrogen as the typical silica surface catalysis recombination reactions. Little NO and other species are found in the gaseous productions. No silicon-containing substance is found in the gaseous products, which means the silica surface plays the role of catalyzer during the recombination reactions. Compared with the gas column model established by Cozmuta et al. [54], the flux boundary conditions employed in this work significantly reduce the generation of unstable reaction products such as O_3_N, ON_3_, ON_4_, ON_5_, and O_2_N_3_ found from the results of Cozmuta et al. [54], indicating the effectiveness and benefits of the flux boundary applied in the work compared with the original gas column model.

**Figure 15:**
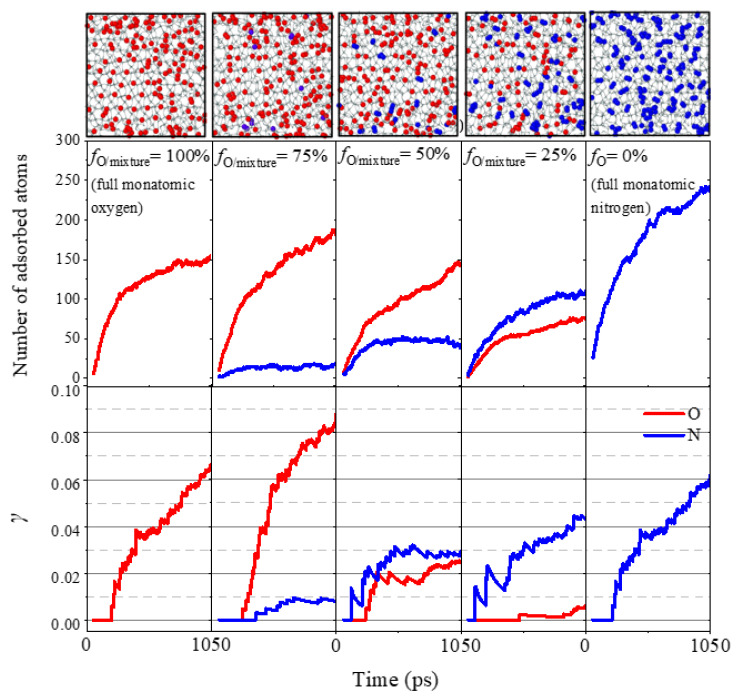
The multicomponent gas effects on the silica surface catalytic characteristics.

To quantify the surface catalysis recombination coefficients of both oxygen and nitrogen for the bicomponent gas mixture bombardment, the transient surface adsorption properties and surface recombination coefficients are presented as shown in Figure 15, associated with the final surface saturation snapshots for each model. It can be found that the corresponding surface catalysis coefficient is not the simple superposition of the incoming gas mixtures: (I) with the decrease in the proportion of O atoms in the high enthalpy gas flow from *f*_O/mixture_ = 75% to 0%, both the number of oxygen atoms adsorbed on the surface and the oxygen catalytic recombination coefficient (0.083, 0.025, 0.006 and 0.0) are decreased, corresponding to an increase in the number of ANs attached to the surface and the nitrogen catalytic recombination coefficient (i.e., 0.009, 0.029, 0.044 and 0.060). (II) When the proportion of O and N atoms in the impinging high enthalpy atomic flow is equal (50%), the number of O atoms adsorbed on the surface is found higher than that of N atoms. This can be explained by Si–O chemical bond energy (~452 KJ/mol) being higher than Si–N chemical bond energy (~355 KJ/mol) [54]. However, the recombination coefficient *γ*_O2_ is lower than *γ*_N2_ due to their intramolecular bond energy differences. (III) For *f*_O/mixture_ = 75%, the AO adsorption rate on the silica surface and the surface recombination coefficient is slightly higher than that of pure AO impinging flow (*f*_O/mixture_ = 100%). This can be explained by the potential reason of denser and more even AO adsorption distribution (intermolecular energy) onto the silica surface under the effect of extra atomistic interactions due to the existence of AN.

## 4. Conclusions

In this study, a GSI model with a flux boundary was established to investigate the surface catalysis recombination mechanisms for silica surfaces under hyperthermal flows. Using the RMD simulation method, the effects of solid surface temperature, gas incident angle, and translational energy on the silica surface catalysis recombination were qualified in the presence of AO, AN, and various AN/AO mixtures. The main conclusions can be drawn as follows:

1. With the increase in the silica surface temperature *T*_s_ from 500 K to 2000 K, the surface coverage fraction of AO becomes greater due to the increased number of active sites on the silica surface, which enhances the percentages of L–H recombination reactions with diffusion recombination mechanisms for molecular oxygen formation.
2. The catalysis recombination coefficient γ under hyperthermal AO impacts is sensitive to the solid surface temperature, the gas angles of attack, and the incidence translational energy. A non-linear increasing pattern of *γ*_O2_ with the increase in AO incident angle *θ*_in_ and translational energy *E*_k_ is observed. There is an optimal relationship between the incident angle and translational energy to achieve the highest surface catalytic recombination rate. High vertical-component kinetic energy may lead AO to directly bounce away from the surface without participating in the surface catalysis recombination reactions.
3. Compared with the surface catalysis under hyperthermal AO impact, the AN surface adsorption fraction shows an inverse trend with the increase in surface temperature, which suggests the potential inadequacy of the traditional proportional-relationship assumption between the surface adsorption concentration and the surface catalysis recombination coefficient for other species, except the AO.
4. For bi-component AO/AN gas mixtures, the corresponding surface catalysis coefficient is not the simple superposition of the effects of individual gases but is affected by both the intramolecular bond energies of O_2_ and N_2_ and intermolecular energies (e.g., Si/N, Si/O), potentially varying the surface adsorption fraction and distribution.

## Figures and Tables

**Table 1:** Parameters of GSI models investigated in the present work.

Type	Model	Surface Temperature, *T*_s_	Incident Angle, *θ*_in_	Incident Translational Energy, *E*_k_	Incident Components, *f*_O/mixture_
**Benchmarking Case**	**1** **(Standard)**	**1000 K**	**90°**	**0.05 eV**	**100%—full AO**
**Set I: Effect of silica surface temperature *T*_s_**	2–4	**500 K, 1500 K, and 2000 K**	90°	0.05 eV	100%—full AO
**Set II: Effect of AO incident angle *θ*_in_**	5–9	1000 K	**15°, 30°, 45°, 60°, and 75°**	0.05 eV	100%—full AO
**Set III: Effect of AO incident translational energy *E*_k_**	10–16	1000 K	90°	**0.01, 0.10, 0.50, 1.00, 2.00, 3.00, and 4.00 eV**	100%—full AO
**Set IV: Effect of *T*_s_, *θ*_in_, and *E*_k_ on silica surface catalysis performance with AN bombardment**	17–20	**500 K, 1000 K, 1500 K, and 2000 K**	90°	0.05 eV	**full AN**
	21–26	1000 K	**15°, 30°, 45°, 60° 75°, and 90°**	0.05 eV	**full AN**
	27–34	1000 K	90°	**0.01, 0.05, 0.10, 0.50, 1.00, 2.00, 3.00, and 4.00 eV**	**full AN**
**Set V: Bicomponent monatomic gas mixture effect**	35–38	1000 K	90°	0.05 eV	**75%** (AO: 75%; AN: 25%), **50%** (AO: 50%; AN: 50%), **25%** (AO: 25%; AN: 75%) and **0%** (AO: 0%; AN: 100%)

**Table 2:** Surface reaction mechanisms.

Reaction	Mechanism
O + (s) → O(s)	Adsorption
N + (s) → N(s)	
O(s) → O + (s)	Desorption
N(s) → N + (s)	
O_2_(s) → O_2_ + (s)	
N_2_(s) → N_2_ + (s)	
NO(s) → NO + (s)	
O + O(s) → O_2_ + (s)	E–R mechanism
N + N(s) → N_2_ + (s)	
N + O(s) → NO + (s)	
O + N(s) → NO + (s)	
O(s) + O(s) → O_2_ + (s)	L–H mechanism
N(s) + N(s) → N_2_ + (s)	
N(s) + O(s) → NO + (s)	

**Table 3:** Comparison of the activation energy for surface catalysis reactions of Models 1–4.

Source	Activation Energy
This work	0.134 ± 0.008 eV
Norman et al. [55]	0.138–0.172 ± 0.012 eV
Balat et al. [23]	0.296 ± 0.019 eV
Dicken et al. [24]	0.153 ± 0.040 eV
Kim and Bourdart [26]	0.166 ± 0.020 eV

## Data Availability

Data available on request.
